# Polygenic risk prediction and *SNCA* haplotype analysis in a Latino Parkinson’s disease cohort

**DOI:** 10.1016/j.parkreldis.2022.06.010

**Published:** 2022-06-18

**Authors:** Douglas P. Loesch, Andrea R.V.R. Horimoto, Elif Irem Sarihan, Miguel Inca-Martinez, Emily Mason, Mario Cornejo-Olivas, Luis Torres, Pilar Mazzetti, Carlos Cosentino, Elison Sarapura-Castro, Andrea Rivera-Valdivia, Angel C. Medina, Elena Dieguez, Victor Raggio, Andres Lescano, Vitor Tumas, Vanderci Borges, Henrique B. Ferraz, Carlos R. Rieder, Artur Schumacher-Schuh, Bruno L. Santos-Lobato, Carlos Velez-Pardo, Marlene Jimenez-Del-Rio, Francisco Lopera, Sonia Moreno, Pedro Chana-Cuevas, William Fernandez, Gonzalo Arboleda, Humberto Arboleda, Carlos E. Arboleda-Bustos, Dora Yearout, Cyrus P. Zabetian, International Parkinson Disease Genomics Consortium (IPDGC), Timothy A. Thornton, Ignacio F. Mata, Timothy D. O’Connor

**Affiliations:** a Institute for Genome Sciences, University of Maryland School of Medicine, Baltimore, MD, USA; b Program in Personalized and Genomic Medicine, University of Maryland School of Medicine, Baltimore, MD, USA; c Department of Medicine, University of Maryland School of Medicine, Baltimore, MD, USA; d Department of Biostatistics, University of Washington, Seattle, WA, USA; e Lerner Research Institute, Genomic Medicine, Cleveland Clinic, Cleveland, OH, USA; f Neurogenetics Research Center, Instituto Nacional de Ciencias Neurologicas, Lima, Peru; g Center for Global Health, Universidad Peruana Cayetano Heredia, Lima, Peru; h Movement Disorders Unit, Instituto Nacional de Ciencias Neurologicas, Lima, Peru; i School of Medicine, Universidad Nacional Mayor de San Marcos, Lima, Peru; j Universidad Nacional del Altiplano, Puno, Peru; k Neurology Institute, Universidad de la República, Montevideo, Uruguay; l Department of Genetics, Facultad de Medicina, Universidad de la República, Montevideo, Uruguay; m Ribãirao Preto Medical School, Universidade de São Paulo, Ribeirão Preto, Brazil; n Movement Disorders Unit, Department of Neurology and Neurosurgery, Universidade Federal de São Paulo, São Paulo, Brazil; o Departamento de Neurologia, Universidade Federal de Ciências da Saúde de Porto Alegre, Porto Alegre, Brazil; p Serviço de Neurologia, Hospital de Clínicas de Porto Alegre, Porto Alegre, Brazil; q Departamento de Farmacologia, Universidade Federal do Rio Grande do Sul, Brazil; r Instituto de Ciências da Saúde, Universidade Federal do Pará, Belém, Brazil; s Neuroscience Research Group, Medical Research Institute, Faculty of Medicine, Universidad de Antioquia (UdeA), Medellín, Antioquia, Colombia; t CETRAM, Facultad de ciencias Medicas, Universidad de Santiago de Chile, Chile; u Neuroscience and Cell Death Research Groups, Medical School and Genetic Institute, Universidad Nacional de Colombia, Bogotá, Colombia; v Veterans Affairs Puget Sound Health Care System, Seattle, WA, USA; w Department of Neurology, University of Washington, Seattle, WA, USA

## Abstract

**Background:**

Large-scale Parkinson’s disease (PD) genome-wide association studies (GWAS) have, until recently, only been conducted on subjects with European-ancestry. Consequently, polygenic risk scores (PRS) constructed using PD GWAS data are likely to be less predictive when applied to non-European cohorts.

**Methods:**

Using GWAS data from the largest study to date, we constructed a PD PRS for a Latino PD cohort (1497 subjects from LARGE-PD) and tested it for association with PD status and age at onset. We validated the PRS performance by testing it in an independent Latino cohort (448 subjects) and by repeating the analysis in LARGE-PD with the addition of 440 external Peruvian controls. We also tested *SNCA* haplotypes for association with PD risk in LARGE-PD and a European-ancestry PD cohort.

**Results:**

The GWAS-significant PD PRS had an area under the receiver-operator curve (AUC) of 0.668 (95% CI: 0.640–0.695) in LARGE-PD. The inclusion of external Peruvian controls mitigated this result, dropping the AUC 0.632 (95% CI: 0.607–0.657). At the *SNCA* locus, haplotypes differ by ancestry. Ancestry-specific *SNCA* haplotypes were associated with PD status in both LARGE-PD and the European-ancestry cohort (p-value < 0.05). These haplotypes both include the rs356182 G-allele, but only share 14% of their variants overall.

**Conclusion:**

The PD PRS has potential for PD risk prediction in Latinos, but variability caused by admixture patterns and bias in a European-ancestry PD PRS data limits its utility. The inclusion of diverse subjects can help elucidate PD risk loci and improve risk prediction in non-European cohorts.

## 1. Introduction

Parkinson’s Disease (PD) is the fastest growing neurological disorder in the world, affecting more than six million individuals [1]. Like all complex disorders, PD etiology is thought to be due to the combination of genetic and environmental risk factors, with common variants of small effect comprising the major component of genetic risk factors [2]. Genome-wide association studies (GWAS) have been used to identify genetic variants that modify disease risk, age at onset, and disease progression. In PD, the largest GWAS effort to date is Nalls et al., 2019 [3], though this study only included European ancestry subjects. Fortunately, diversity in PD research is increasing: Foo et al., 2020 have conducted the largest study of PD patients with East Asian ancestry [4] and our group has conducted the largest study of South American PD patients [5].

Outside of risk variant and disease-gene discovery, a primary use of GWAS is to generate summary statistics for the purpose of risk prediction using polygenic risk scores (PRS). A PRS is the linear summation of disease risk variants weighted by their effect size and has been shown to improve disease risk prediction [6]. The PRS model has been applied to an increasing number of diseases with the eventual goal of risk stratification followed by clinical interventions [6]. In PD, Nalls et al. evaluated PRS models that demonstrated promise for PRS-based PD risk prediction [3].

However, transferring a PRS generated using GWAS from one population to another with a different ancestry background [7,8] is often suboptimal. It is thought that this lack of portability is primarily due to either differences in allele frequencies or linkage disequilibrium (LD) patterns [9,10]. However, since a PRS depends on accurate effect size estimates, very large sample sizes are needed to achieve adequate out-of-sample prediction [11]. Due to the persistent lack of diversity in GWAS data, large sample sizes are typically only available for European or East Asian-ancestry subjects [9]. This is a major challenge for the clinical implementation of PRS-based risk prediction [10].

In PD, we also see the drop in performance when translating PRS across populations. Foo et al. applied a PRS based on the Nalls et al. GWAS-significant variants to PD patients from East Asia; the performance of the PRS lagged behind that of European cohorts, though this was remedied via the inclusion of Asian-specific data [4]. Here, we construct PRS using summary statistics from Nalls et al., 2019 [3] and tested it in our Latino case-control cohort from the Latin American Research Consortium on the Genetics of Parkinson’s Disease (LARGE-PD) [5,12]. We also explore the haplotype structure of rs356182 near *SNCA*, a major component of the PD PRS and thought to be a key gene in PD etiology [13], across ancestrally diverse populations.

## 2. Methods

### 2.1. Cohort descriptions

The LARGE-PD cohort consists of 807 PD cases and 690 controls from Uruguay, Peru, Chile, Brazil, and Colombia, with 1481 samples that feature complete age and sex records after quality control. PD patients were evaluated by a local movement disorder specialist using the UK PD Society Brain Bank clinical diagnostic criteria (UKPDSBB) [14]. Individuals who did not exhibit neurological symptoms were selected as controls. All participants provided written informed consent according to their respective locale’s national requirements. For validating the PD PRS performance in Latinos, we utilized a cohort of 448 Latinos (223 controls and 225 cases) provided by the International Parkinson Disease Genomics Consortium (IPDGC) [15]. The IPDGC also provided 715 PD subjects and 1731 controls of European ancestry for our analysis of *SNCA* haplotypes (IPDGC-EUR). We leveraged 440 subjects from a Peruvian tuberculosis cohort downloaded from dbGaP with IRB approval (Luo et al., 2019; subjects had a General Research Use consent) to use as additional controls to further evaluate our PRS models [16]. We utilized 1000 Genomes Project (1KGP) [17] and Peruvian Genome Project [18] (PGP) samples as references in our haplotype analysis and to explore the relationship between the PD PRS and inferred ancestry. All genotyped cohorts were imputed using the TOPMed Imputation Server [19] and filtered with a minimum imputation R^2^ of 0.3. We performed relationship inference using the KING software [20]; unless otherwise specified, close relatives were defined as having a kinship coefficient greater than 0.0884 (2nd degree). See Supplementary Table 1 and supplementary methods for further description of all cohorts utilized in this study.

### 2.2. PRS estimation and evaluation

We utilized summary statistics from Nalls et al., 2019 [3]; we lifted the positions to hg38 using UCSC LiftOver utility. After removing sites that were strand ambiguous (i.e. CG/AT), we calculated PRS using R and PLINK 1.9 [21] with 77 independent GWAS-significant PD risk variants. To construct a full-summary statistics PRS (PRS-full), we selected variants with a minor allele frequency (MAF) of 5% and used PRSice-2 [22] with its default settings (R^2^ threshold of 0.1 and a 250 kilobase window) to perform pruning and thresholding, resulting in a PRS estimated using 1040 variants. This emulated the approach used by Nalls et al. [3], though by training it in LARGE-PD we were able to obtain the optimal p-value threshold given the remaining parameters. We evaluated all PRS models using R. We validated both PRS models using an independent Latino PD cohort from the IPDGC. We also repeated the above analyses after incorporating external Peruvian controls from the Luo et al. cohort with LARGE-PD (see supplementary methods).

We visualized the PRS distribution in LARGE-PD and the external Peruvian controls after clustering by principal component (PC). The ancestry of each cluster was inferred using ADMIXTURE [23]. We then explored the relationship of admixture with the PD PRS in 1KGP Latino populations by utilizing the ancestry proportions estimated with ADMIXTURE and obtaining correlations between ancestry proportions and the PD PRS using Pearson’s method. We then characterized the PD PRS distribution in every 1KGP population [24], assessing differences in distributions between populations using the Wilcoxon rank-sum test. See supplementary methods for complete description of methods.

### 2.3. Age at onset analysis of the GWAS-significant PRS

We assessed the impact of the PRS on the age at onset (AAO) of PD using a filtered dataset consisting of the unrelated LARGE-PD subjects with an AAO after the age of 18. We generated Kaplan–Meier curves of the GWAS-significant PRS stratified by quintile. For the event, we used the diagnosis of PD; for time to event, we used age of onset where available and age at analysis for controls and cases lacking age of onset data. We then performed a Cox regression analysis; we again stratified the PRS by quintile and adjusted for sex, the first 10 PCs, and recruitment site. We performed all analyses using the survival package [25] in R.

### 2.4. SNCA Haplotype analysis

We generated a joint dataset comprised imputed LARGE-PD data along with PGP, 1KGP, and IPDGC-EUR sequence data, resulting in the intersection of 108,118 variants spanning the *SNCA* locus. The merged dataset was then jointly phased using Beagle 5.0 on default settings [26]. We utilized PLINK’s haplotype block procedure to estimate haplotype blocks in this region. We then extracted a region corresponding to the LARGE-PD Peruvian haplotype block containing rs356182 with a total of 52 intersecting variants with a minimum MAF of 5%. We constructed a haplotype network using POPArt [27] and the TCS method [28].

For LARGE-PD and IPDGC-EUR data, we tested haplotypes with a frequency higher than 1% in each respective cohort for association with PD risk. With LARGE-PD, we utilized the unrelated subset and adjusted for age, sex, recruitment site, and the first 10 PCs. With the IPDGC European-ancestry data, we adjusted for age, sex, cohort, and the first five PCs. Multiple testing correction was applied by adjusting for the number of haplotypes tested in each cohort. Haplotypes with a p-value less than 0.05 were then evaluated using a likelihood ratio test to assess whether the addition of the haplotype demonstrates significant improvement over a model including all covariates and the rs356182 genotype status of each subject.

## 3. Results

### 3.1. PD PRS evaluation and validation

**Fig. 1.**
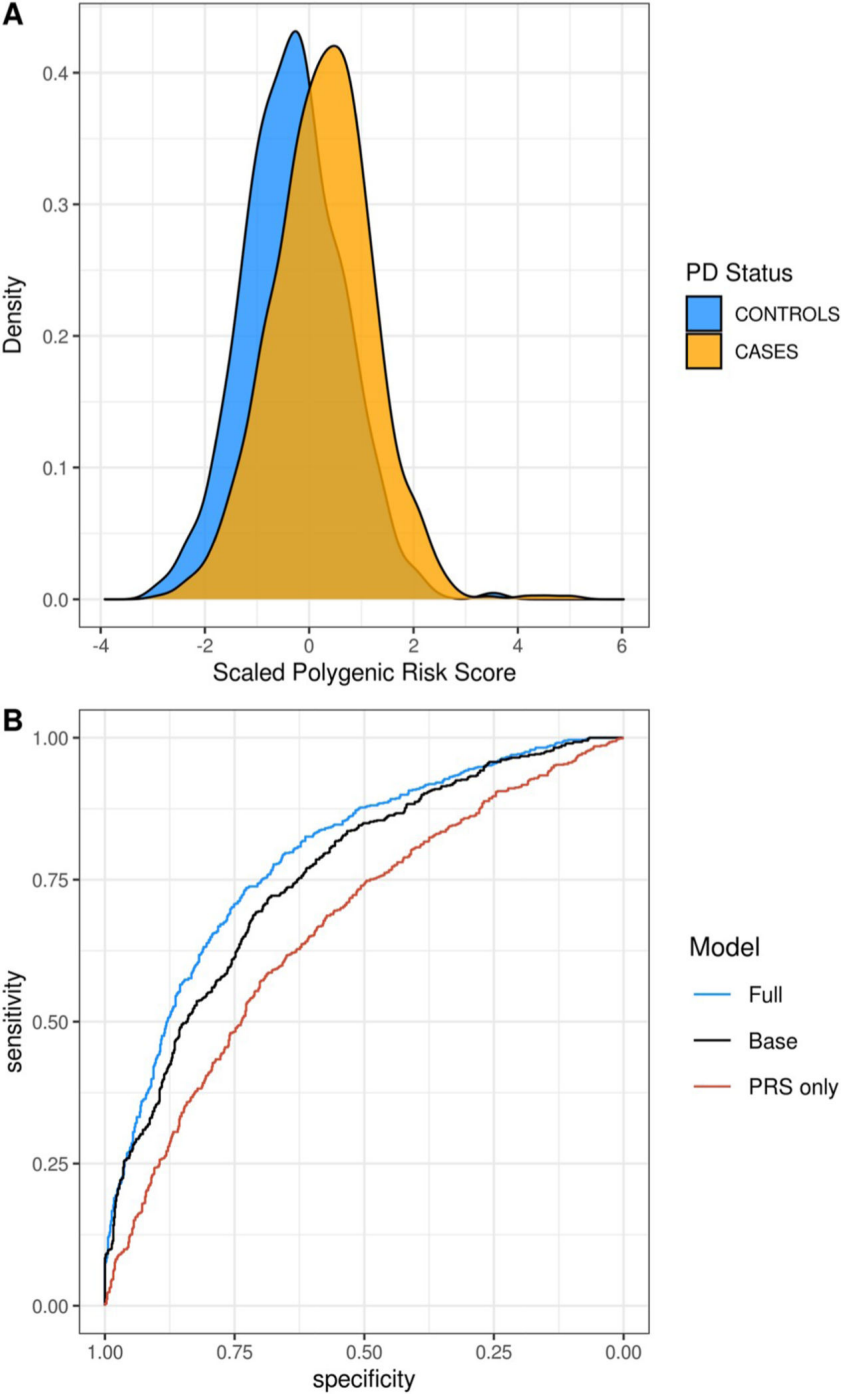
PD PRS prediction in LARGE-PD. **A:** Distribution of the PD PRS constructed using GWAS-significant variants in LARGE-PD cases versus controls. **B:** Receiver-operator curve (ROC) of full model including covariates (age, sex, PCs 1–10, recruitment site) and the GWAS-significant PD PRS (blue), ROC of base model including only covariates (black), and ROC of model including only the GWAS-significant PD PRS (orange). The addition of the GWAS-significant PRS to create the full model improved the AUC by 4.3% over the base model without the PRS (p-value of 1.03 × 10^− 6^, Delong’s test). All models shown in 1B include only data from LARGE-PD. (For interpretation of the references to color in this figure legend, the reader is referred to the Web version of this article.)

We found the GWAS-significant PD PRS to be highly associated with PD status (p-value = 1.91 × 10^−18^) and it explained 2.2% of trait variance on the liability scale (Table 1). The PRS achieved partial seperation of cases and controls (Fig. 1A). When stratifying the PRS by quintile, the highest quintile had an odds ratio of 5.38 (95% CI: 3.78–7.67) when compared to the lowest quintile (Supplementary Fig. 1A). Using only the PRS to predict PD risk, the area under the receiver-operator curve (AUC) was 0.668 (95% CI: 0.640–0.695; Fig. 1B), with a sensitivity of 71.3% and a specificity of 52.1%. The addition of the GWAS-significant PD PRS to a model including all covariates (age, sex, recruitment site, and PCs 1–10) improved the AUC by 4.3% over the base model (p-value of 1.03 × 10^−6^). The PRS using the full GWAS summary statistics (PRS-full) resulted in the inclusion of 1040 variants and had an overall AUC of 0.676 (95% CI: 0.649–0.704), with a sensitivity of 69.8% and a specificity of 53.1%. The AUCs of the GWAS-significant and full summary stat models were not significantly different (p-value = 0.44).

The predictive performance of the PD PRS was remarkable as LARGE-PD is a Latino cohort with a mean European ancestry of only 47% [5]. Also contrary to expectations the performance of the GWAS-significant PRS was driven by Peruvian subjects (Supplementary Fig. 1B), who are predominantly of Native American ancestry [18]. This result was robust to removing close relatives, down-sampling Peruvian PD cases and excluding subjects who are outliers by ancestry (Table 1). After adding Peruvian controls from an external study [16], the AUC of the GWAS-significant PRS dropped to 0.632 (95% CI: 0.607–0.657), with a specificity of 31.9% and a sensitivity of 83.4% (Supplementary Fig. 1B). Furthermore, the variance explained on the liability scale was only 1.5%, though this could be partially attributed to the choice of covariates and the use of external controls. While we sought to minimize array differences through imputation and quality control (see supplementary methods), the external controls were nevertheless not screened for PD. Though the AUC is substantially lower with the inclusion of the external controls, the AUCs of models with and without the external controls were not significantly different (p-value = 0.08), though this could be attributed to sample size limitations.

To validate the GWAS-significant PRS and the PRS-full, we tested both models in an independent cohort of 448 Latinos. The GWAS-significant model had an AUC of 0.665 (95% CI: 0.604–0.705) with a sensitivity of 61.3% and a specificity of 60.0% (Table 1). For the PRS-full, the AUC was 0.662 (95% CI: 0.612–0.712), though only 651 of the variants were imputed at a sufficient level across both genotyping chips used (Supplementary Fig. 2). Again, these results were robust to the removal of relatives up to the 2nd degree.

### 3.2. PD PRS distribution in LARGE-PD and 1KGP

**Fig. 2.**
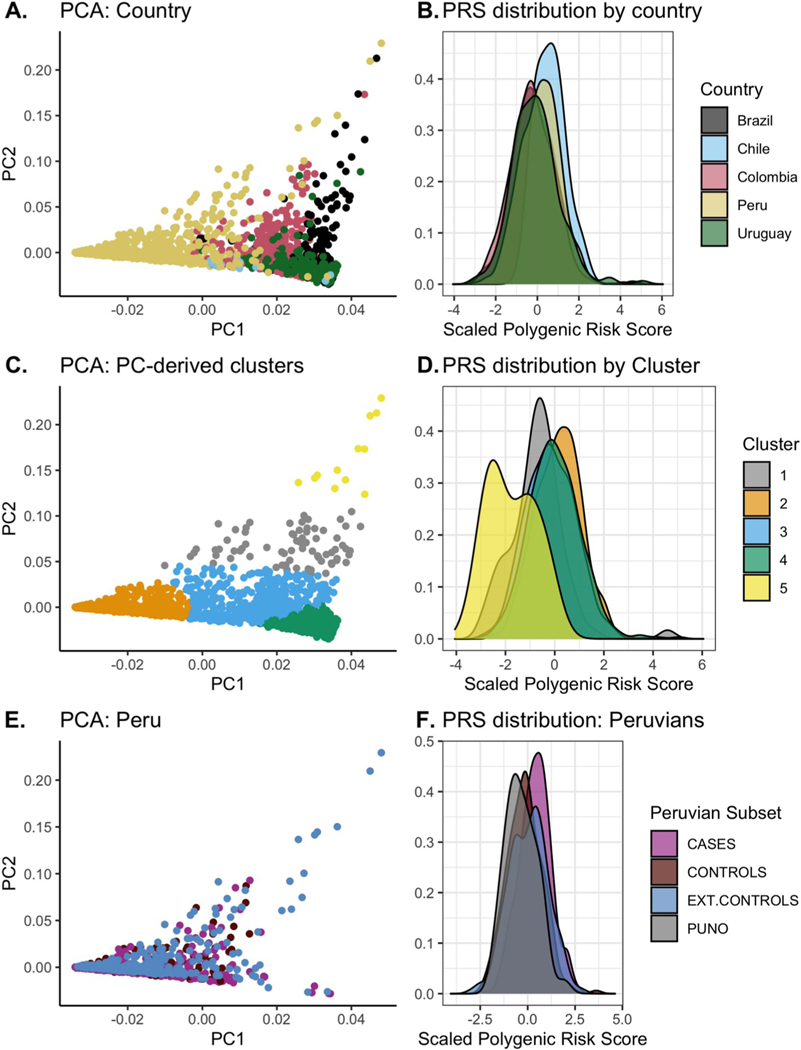
PD PRS distribution in LARGE-PD and external controls. Principal components (PCs) of LARGE-PD plus external controls and density plots of the PD PRS distribution. **A:** Plot of PC 1 versus PC 2 colored by country of origin. **B:** PRS distribution colored by country of origin. **C:** Plot of PC 1 versus PC2 colored by PC-derived clusters using k-means clustering. **D:** Distribution of the PD PRS colored by PC-derived clusters. We used ancestry proportions estimated by ADMIXTURE to characterize clusters. We observed that the African-ancestry cluster (blue) was shifted to the left, the European-ancestry cluster (silver) was centered on zero, and the Native American cluster (orange) was shifted to the right. **E:** PCA plot of subjects from Peru. Subjects are classified as being cases, controls, from Puno (all controls), or external Peruvian controls. **F:** PD PRS distribution in Peru. (For interpretation of the references to color in this figure legend, the reader is referred to the Web version of this article.)

The variability of models using the PD PRS can be visualized by examining the PD PRS distribution by country (Fig. 2 A and B). Excluding Chilean subjects due to sample size, subjects from Peru had the highest mean PRS (mean [SD]: 0.18 [0.55]) while samples from Colombia had the lowest (mean [SD]: − 0.04 [0.62]). This was not attributable to case-control ratio, as the mean PRS was not significantly correlated with the proportion of cases (p-value = 0.75). However, the first four PCs were all significantly correlated with the PD PRS (p-value < 0.05). When clustering samples by PC, the PD PRS distributions reflect the ancestral compositions of the clusters, with inferred African clusters shifted to the left of zero and inferred Native American clusters shifted to the right (Fig. 2C and D). This same pattern is observed when restricting to controls only (Supplementary Fig. 3). The PD PRS distribution varied among our samples from Lima, Puno, and the external controls from Lima. (Fig. 2 E and F).

The 1KGP includes Latinos from Peru, Mexico, Puerto Rico, and Colombia. These individuals are admixed with varying contributions from African, European, and Native American ancestral populations (see supplementary figure 4 A). In 1KGP Latinos, the PD PRS is positively correlated with inferred Native American ancestry (Pearson’s R: 0.19, p-value: 0.0004) and negatively correlated with both European (Pearson’s R: −0.11, p-value: 0.03) and African ancestry (Pearson’s R: −0.30, p-value: 1.4 × 10^−8^; supplementary Fig. 4 B–D).

To highlight the variability in PD risk loci across diverse ancestral populations, we calculated a GWAS-significant PD PRS for every 1KGP subject [17,24]. The PD PRS was lowest in African populations and highest in East Asian populations (Supplementary Fig. 5). For each non-European population, we assessed the difference in PD PRS distribution compared to Europeans using the Wilcoxon Rank-sum test (Supplementary Table 2). The PD PRS distribution significantly differed from European-ancestry samples for every other global population. Differences in the PRS distribution is likely being mediated by population-specific differences in allele frequencies. In particular, variants conferring positive disease risk are demonstrably lower in frequency in African populations compared to European populations as has been previously noted [29] (p-value = 0.001, Chi-square test with 1 degree of freedom; Supplementary Fig. 6). We also estimated a PRS using the 71 risk SNPs in common across every available Peruvian cohort (Supplementary Table 3). LARGE-PD Peruvian controls have a lower mean PD PRS compared to Peruvian subjects from 1KGP (0.54 versus 0.58) or the PGP (0.68). When stratifying the PGP by sub-population, we observed a fair amount of heterogeneity, with the PRS ranging from a mean of 0.58 (the Uros) to 0.88 (the Chopccas).

### 3.3. Age at onset analysis

To evaluate the impact of the PD PRS on disease onset, we generated Kaplan-Meier curves and performed Cox regression using the age at analysis for controls and age at onset for cases. We stratified the GWAS-significant PRS by quintile and found that the AAO decreased when comparing the highest quintile to the lowest (Supplementary Fig. 7). In our Cox regression model, the highest quintile had a hazard ratio (HR) of 2.29 (95% CI: 1.79–2.93; p-value: 3.41 × 10^−11^). We also repeated the analysis using only cases, with the PRS still being significantly associated with AAO, though the effect is attenuated (HR: 1.45, 95% CI: 1.14–1.86, p-value: 0.003; Supplementary Table 4).

### 3.4. SNCA Haplotype analysis

**Fig. 3.**
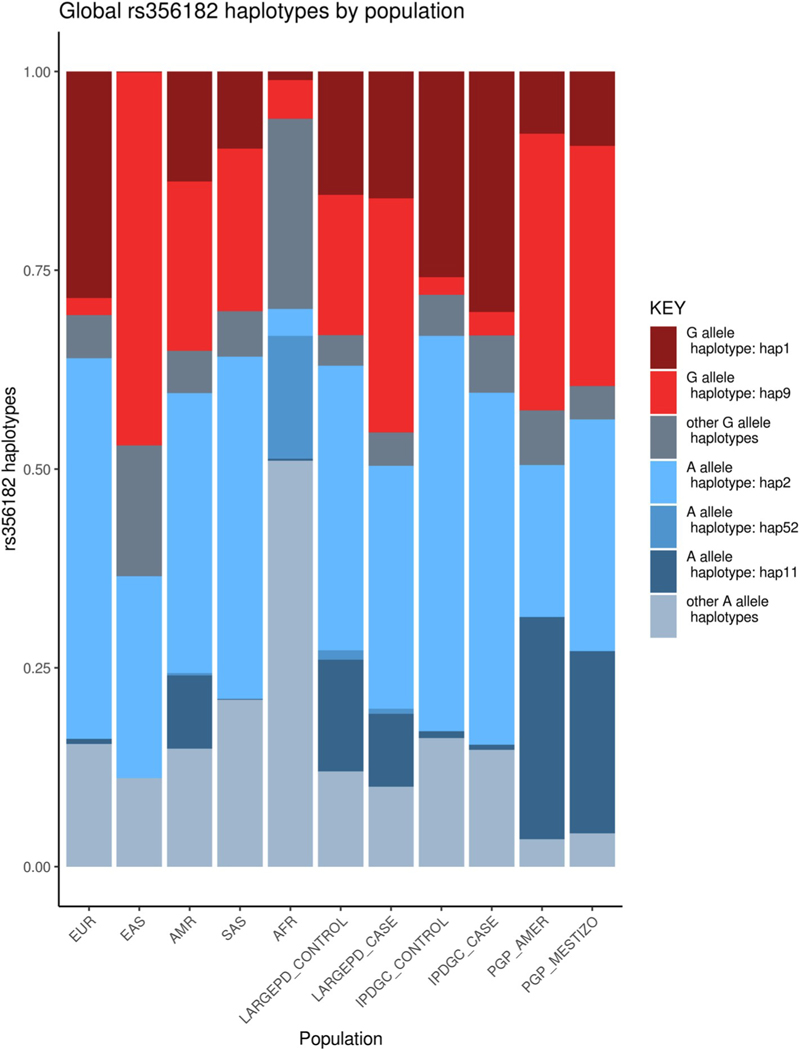
rs356182 Haplotypes by population. Haplotypes of the *SNCA* locus centered on rs356182 consisting of phased genotypes from the 1KGP, LARGE-PD, and an IPDGC PD cohort of European descent. Haplotypes shown here are the most common haplotypes by 1000 Genomes population and PD case-control status. Note the same A haplotype is shared across populations, but the G haplotype exhibits greater population specificity.

The variant rs356182 at the *SNCA* locus explains the largest proportion of trait variance out of the variants included in the GWAS-significant PD PRS (Supplementary Table 5). For most global populations, the rs356182 haplotype block is small due to recombination (Supplementary Table 6). Within the PD cohorts, the largest haplotype blocks were consistently found in Peruvian populations. We extracted the 33.6 kilobase region corresponding to the block found in the Peruvian subset of LARGE-PD and categorized the haplotypes based on their rs356182 allele status (Fig. 3). The same A-allele haplotype is the most common in all out-of-Africa populations in our joint dataset, while the G-allele haplotype appears to be more population specific. We constructed a haplotype network and found that the most common G-allele haplotype in East Asians (hap9) and the most common G-allele haplotype in Europeans (hap1) are separated by several intermediary haplotypes and only share 14% of their alleles (Supplementary Figs. 8 and 9).

We tested rs356182 haplotypes for association with PD in LARGE-PD and the IPDGC-EUR cohort (Supplementary Tables 7 and 8). Overall, tested haplotypes had an 87% concordance between the direction of effect and rs356182 allele status. In LARGE-PD, three haplotypes were nominally associated with PD status: hap6 (p-value = 0.006), hap9 (p- value = 4.47 × 10^−6^), and hap11 (p-value = 6.04 × 10^−9^); all three haplotypes remained significant after adjusting for multiple tests (adjusted p-value < 0.05). In IPDGC-EUR, two haplotypes were nominally associated with PD: hap1 (p-value = 0.01) and hap2 (p-value = 1.75 × 10^−4^). After correcting for multiple testing, hap2 remained statistically significant. We then evaluated whether the addition of haplotype information significantly improvement a model that included rs356182 allele status. In LARGE-PD, hap6 and hap11 were nominally significant (p-value 0.039 and 2.65 × 10^− 5^, respectively) while hap9 was not (p-value 0.076). After correcting for multiple testing, only hap11 remained statistically significant. In the IPDGC-EUR, hap1 and hap2 were not nominally significant (p-values 0.29 and 0.15, respectively). In aggregate, these results indicate that rs356182 is the source of the PD risk conferred by these haplotypes.

## 4. Discussion

Polygenic risk prediction has the potential to identify individuals at higher risk of developing disease who could benefit from interventions and increased monitoring. However, PRS predictive performance depends on GWAS with large sample sizes which are generally only available in European-ancestry cohorts and, to a lesser extent, East Asian ancestry cohorts. The performance of PRS derived from such datasets generally suffers when applied to individuals from a different ancestral background, leading to inaccurate or even biased estimates of disease risk [7,8,10]. In addition, a PRS derived from one ancestry can exhibit shifts in distribution across ancestries that are not necessarily concordant with population-level disease risk. Interpreting and ultimately rectifying these shifts is an area of ongoing research. For PD, the sample size is now sufficiently large that future clinical application of the PD PRS is plausible, though the European bias likely limits its utility.

In LARGE-PD, a Latino PD cohort, we found that the PD PRS constructed using GWAS-significant data performed surprisingly well with an AUC of 0.668, outpacing the AUC obtained in European (0.651) and East Asian cohorts (0.602) when utilizing the Nalls et al. GWAS-significant PD summary statistics [3,4]. This result runs counter to the bulk of the PRS literature; predictive performance should be worse when applying a PRS across ancestries. While it is possible that these GWAS-significant variants might play an outsized role in the etiology of PD in Latinos, a more parsimonious explanation is that bias in the GWAS summary statistics, together with the complex composition of the LARGE-PD cohort, contribute to the performance we observed. We do find evidence that the PD PRS exhibits population-wide shifts in distribution. In both LARGE-PD and 1KGP Latinos, we found that the PD PRS exhibits a bias by ancestry where individuals with high Native American ancestry tended to have a higher PD PRS, while individuals with high African ancestry were more likely to have a lower PD PRS (Fig. 2, Supplementary Figs. 4–6). In 1KGP, we found that the PD PRS distribution significantly differs from that of Europeans in all other populations (p-value < 0.05, Wilcoxon). Shifts in a PRS distribution by ancestry has been previously characterized in other traits but are not always concordant with known disease prevalence rates [8]. Since GWAS summary statistics used for PRS estimation generally suffer from a European-ascertainment bias, shifts in PRS distribution do not necessarily reflect true genetic risk and scores are not comparable across ancestries [30]. Only by increasing representation in GWAS data can the relationship between polygenic risk and ancestry be elucidated. Nevertheless, characterizing the behavior of PRS in non-Europeans when using currently available GWAS data is critical, particularly as the field considers applying PRS to the clinic.

In LARGE-PD, PD cases had a higher mean Native American ancestry than controls which could be contributing to the surprisingly strong performance of the PD PRS as measured by AUC of the PRS alone. This is likely due to population stratification aligning with the by-ancestry bias of the PD PRS. In the case of Peruvian subjects, the AUC remained high even when we down-sampled Peruvian cases and when we fit models with only Peruvian subjects (Table 1). However, when we included external Peruvian subjects as controls, we saw a reduction in the model’s AUC. We found that Peruvian LARGE-PD controls from Lima and Puno have a lower mean PRS than any other Peruvian subpopulation. This suggests that LARGE-PD controls have been sampled from the lower end of the PD PRS distribution in Peru, either purely due to chance or because they belong to a subpopulation with a lower frequency of PD risk alleles used in the PRS. Together, these results suggest that the PD PRS is impacted by population history and that PRS performance metrics without the use of PCs as covariates were likely inflated due to the correlation of the PD PRS with ancestry. Indeed, when comparing variance explained on the liability scale when accounting for covariates, the variance explained by the GWAS-significant PRS in LARGE-PD was lower (2.2%) than that estimated in a European-ancestry cohort (3.5%) [3], in contrast to the AUC of the PRS. Reporting the AUC of the PRS alone without correcting for population structure appears ill-advised, particularly in the presence of admixture.

Despite the challenges, the use of a PD PRS for risk prediction in Latinos certainly has potential. In all scenarios we tested, the PD PRS achieved a degree of separation between cases and controls (Table 1). In addition, PD cases with a PD PRS in the highest quintile had a hazard ratio of 1.45 compared to PD cases in the lowest quintile, demonstrating that the PD PRS contributed to the modification of disease course. Due to the bias in the PD PRS distribution, care needs to be taken when interpreting results and the inclusion of covariates are necessary to mitigate confounding. As demonstrated by LARGE-PD, the admixture patterns in a cohort can have a strong impact on the PRS performance. Before it can be used in the clinic, the challenges of translating the PD PRS across populations will need to be addressed through the inclusion of diverse GWAS data and improved methods development.

As the GWAS variant with the largest expected effect size among common variants, rs356182 in *SNCA* contributes to a significant proportion of the variance explained in the PD PRS. Shifts in rs356182 frequency or exclusion of this SNP due to poor genotyping can have a large impact on the predictive accuracy of the PD PRS. Consequently, we conducted a haplotype analysis at the *SNCA* locus centered on rs356182. Haplotype blocks were generally small in most populations and rs356182 was not well tagged (defined as an r^2^ > 0.8) in any non-PD cohort due to recombination. An examination of the most frequently seen haplotypes in this region, though, reveals global patterns. Nearly every population shares the same common A-allele haplotype, while the most common G-allele haplotype in European-ancestry individuals differs from East Asian, South Asian, and Native American individuals (Fig. 3). In LARGE-PD, the non-European G-allele haplotype (hap9) was robustly associated with PD status, while in a European-ancestry cohort, the European G-allele haplotype (hap1) was nominally significant. These two haplotypes only share 14% of their alleles (Supplementary Fig. 9) and are likely independently derived from the African A haplotype, which points towards rs356182 as driving the PD risk conferred by these haplotypes. Further supporting this conclusion, in both cohorts, the directions of effect for the *SNCA* haplotypes were overwhelmingly concordant with their rs356182 allele status and the inclusion of four of five nominally significant haplotypes did not significantly improve models with rs356182 genotype status after correcting for multiple tests. Since rs356182 has replicated across three populations (East Asian, European, and Latino) [3–5] and the structure of the underlying haplotypes differ substantially, a functional role for rs356182 appears likely as has previously been suggested through bioinformatic predictors and functional data [13].

### 4.1. Limitations and strengths

Our study was limited by sample size, particularly on the country or sub-population level. The predictive performance of the PD PRS could differ in larger Latino populations, particularly if the ancestral composition differs from that of LARGE-PD. In addition, our use of external samples as controls could introduce a degree of error due to not being explicitly screened for PD status. Finally, the summary statistics used to construct PRS for this study were generated using only European-ancestry data. As more diverse PD GWAS data becomes available, PD PRS estimation in non-European cohorts will likely improve. Despite these limitations, we show both the potential and shortcomings of utilizing a European-ancestry PD PRS in non-European cohorts and highlight the bias in the PD PRS by ancestry. We also provide orthogonal evidence suggesting that rs356182 is a functional variant, again demonstrating the value of including diverse subjects in PD research.

## Supplementary Material

Supplement

## Figures and Tables

**Table 1:** PD PRS predictive performance in Latino PD cohorts.

COHORT	SUBSET	PRS TYPE	PVALUE	R2	PSEUDO R2	AUC (95% CI)	ACC (BAL)	SPEC	SENS

LARGE-PD	ALL	GWAS	1.91 × 10^−18^	0.022	0.058	0.668 (0.640–0.695)	0.625 (0.617)	0.521	0.713
	UNREL - 2nd Degree	SIGNIF	1.68 × 10^−18^	0.023	0.059	0.668 (0.640–0.696)	0.627 (0.619)	0.518	0.721
	UNREL - 3rd Degree		1.08 × 10^−17^	0.022	0.057	0.666 (0.638–0.694)	0.631 (0.622)	0.520	0.725
	OUTLIERS		3.36 × 10^−18^	0.021	0.055	0.666 (0.638–0.694)	0.626 (0.619)	0.527	0.710
	DOWN SAMPLED		6.32 × 10^−16^	0.022	0.057	0.664 (0.634–0.694)	0.632 (0.626)	0.535	0.717
	PERU ONLY		8.60 × 10^−11^	0.028	0.068	0.675 (0.635–0.716)	0.670 (0.618)	0.379	0.857
	PERU EXCL.		1.48 × 10^−08^	0.016	0.044	0.629 (0.589–0.668)	0.599 (0.594)	0.490	0.697
	ALL	FULL	2.37 × 10^−22^	0.028	0.072	0.676 (0.649–0.704)	0.621 (0.615)	0.531	0.698
		SUM							
		STATS							
LARGE-PD + EXTERNAL CONTROLS	ALL	GWAS	2.18 × 10^−18^	0.015	0.038	0.632 (0607–0.657)	0.620 (0.577)	0.319	0.834
	UNREL	SIGNIF	2.31 × 10^−19^	0.015	0.039	0.635 (0.608–0.660)	0.623 (0.580)	0.326	0.833
	PERU ONLY		6.25 × 10^−11^	0.012	0.029	0.645 (0.612–0.677)	0.639 (0.557)	0.229	0.885
NEUROX + NEUROC	ALL	GWAS	NA	NA	NA	0.655 (0.604–0.705)	0.596 (0.596)	0.583	0.609
	UNREL	SIGNIF	NA	NA	NA	0.654 (0.603–0.706)	0.585 (0.582)	0.507	0.656
	ALL	FULL	NA	NA	NA	0.662 (0.612–0.712)	0.607 (0.607)	0.601	0.613
	UNREL	SUM	NA	NA	NA	0.657 (0.606–0.708)	0.608 (0.606)	0.550	0.66
		STATS							

COHORT: cohort label. SUBSET: subpopulation label from cohort. PRS TYPE: type of model used (either GWAS significant SNPs or the full summary stats). PVALUE: p-value of the PRS in a logistic regression model. R2: variance explained on the liability scale. PSEUDO R2: Nagelkerke’s Pseudo R^2^. AUC (95% CI): area under the receiver-operator curve and 95% confidence intervals. ACC (BAL): accuracy and balanced accuracy of PRS alone. SPEC: specificity of the PRS alone. SENS: sensitivity of the PRS alone. The p-value, R^2^ on the liability scale, and pseudo-R^2^ were estimated in models including covariates. Accuracy, balanced accuracy, specificity, and sensitivity were estimated in models using only a PRS.

## Data Availability

Code used for this project can be accessed at www.github.com/dloesch/LARGEPD_PRS. 1000 Genomes Project sequence data can be found at https://www.internationalgenome.org/. International Parkinson’s Disease Genomics Consortium (IPDGC) data is available here https://pdgenetics.org/resources and additional inquiries regarding IPDGC data can be made at https://pdgenetics.org/contact. Peruvian Genome Project data is available through the European Genome-Phenome Archive (EGA): https://ega-archive.org/datasets/EGAD00001007082. Data from Luo et al. is available on the database of Genotypes and Phenotypes (dbGaP) with accession number phs002025.v1. p1. LARGE-PD genotype data will be uploaded to dbGaP for recruitment sites that have completed the dbGaP certification process. Summary statistics for the full LARGE-PD cohort are currently available in the PD GWAS browser: https://pdgenetics.shinyapps.io/GWASBrowser/.
