# Supplementary material for: Polygenic risk prediction and SNCA haplotype analysis in a Latino Parkinson’s disease cohort
Source: Parkinsonism Relat Disord. Author manuscript; Available in PMC 2023 Sep 1. (PMC10112543; doi:10.1016/j.parkreldis.2022.06.010)
Supplement: Supplement [file NIHMS1891045-supplement-Supplement.docx]

**Supplemental Methods**

*LARGE-PD Cohort Description*

1,504 LARGE-PD individuals from Uruguay, Peru, Chile, Brazil, and Colombia have genotype data available. Samples were genotyped using the Multi-ethnic genotyping array (MEGA) chip from Illumina [1]. Genotyped subjects have a mean age of 59.3 (± 13.9) years; 44.3% are male and 55.7% female. Overall, the analysis dataset consists of 807 PD cases and 690 controls after quality control, with 1481 samples that feature complete age and sex records (1447 unrelated to the 2nd degree). PD patients were evaluated by a local movement disorder specialist using the UK PD Society Brain Bank clinical diagnostic criteria (UKPDSBB) [2]. Individuals who did not exhibit neurological symptoms were selected as controls. All participants provided written informed consent according to their respective locale’s national requirements. A complete description of LARGE-PD, including ancestry composition, ascertainment, quality control, and imputation, can be found in Loesch et al. 2020 [3].

*Additional PD Cohorts Description*

See **supplementary table 1** for the description of all cohorts used in this study. For validating the PD PRS performance in Latinos, we utilized a cohort of Latinos provided by the International Parkinson Disease Genomics Consortium (IPDGC) [4]. These subjects were identified as Latinos based on principal components analysis and were excluded from the primary IPDGC GWAS. The first cohort, which will be referred to as NeuroX_C, consists of 448 subjects, with 223 controls and 225 cases (49.55% male, age information unavailable; 431 unrelated). Genotyping was done using neurogenerative disease-specific genotyping chips: 155 of the samples were genotyped using the NeuroX [5] chip; the remainder were genotyped using the NeuroChip (NeuroC) [6].

The IPDGC also provided 715 PD subjects and 1731 controls of European ancestry for our analysis of ­*SNCA* haplotypes, which we will refer to as IPDGC-EUR. All IPDGC-EUR subjects have undergone whole genome sequencing, have a mean age of 73 years (±18.4) and are 54.2% male.

*Non-PD Cohort Description*

We utilized the unrelated subset of the high-coverage 1000 Genomes Project data generated by the New York Genome Center[7] as references in our haplotype analysis and to estimate the PD PRS distribution across ancestral populations. We also utilized all sequenced Peruvian Genome Project [8] samples as additional Native American references in our haplotype analysis.

We used 440 subjects over the age of 50 (429 unrelated) from a Peruvian tuberculosis (TB) cohort from Luo et al. to use as additional controls in order to evaluate the robustness of our PRS models.[9] These samples have a mean age of 62.65 (SD: 9.13) years and are 46.4% male. They were genotyped using the Affymetrix LIMAArray, a custom array with 720,000 SNPs. [9] For this study, we will refer to this cohort as Luo_TB. This cohort was downloaded from dbGaP under the supervision of an Institutional Review Board. All subjects have a General Research Use (GRU) consent.

*Imputation of Genotyped Samples*

The NeuroX, NeuroC, and Luo_TB cohorts were filtered for 10% sample and site missingness, a Hardy-Weinberg exact (HWE) test p-value < 1x10^-6^, keeping only biallelic SNPs. We then imputed each cohort separately using the TOPMed Imputation Server hosted by the University of Michigan [10]. The imputation pipeline employs Eagle2 for phasing, Minimac4 for imputation, and filters results by an R^2^ of 0.3. The TOPMed Imputation server has been shown to improve imputation for Latino populations and is currently the best publicly-available option [10,11].

*PRS estimation and evaluation*

Custom R code was used to generate the GWAS-significant PD PRS. In addition, a leave-one-out procedure was conducted where each SNP was iteratively removed from the PD PRS. For the PD PRS using the full GWAS from Nalls et al. [12] summary statistics, PRSice-2 was utilized [13] using the same parameters of Nalls et al. [12] PRSice-2 iterates over a range of p-values to select the parameters that explain the most trait variance. PRSice-2 calculates the observed variance using Nagelkerke’s pseudo R^2^, converting to the liability scale using a given prevalence, in this case 0.5%. It is recommended to utilize a LD-reference panel that matches the population used to generate the summary statistics, so we utilized 500 European samples from the 1000 Genomes Project[14] as the LD reference panel.

Both the custom code and PRSice-2 calculated the PRS using the following equation:

$$PRS_{i} =\sum_{j=1}^{M} \frac{\beta_{j}X_{ij}}{M}$$

where the PRS of individual *i* is calculated by taking the summation of the individual’s genotype (0,1,2) of SNP *j* weighted by its corresponding effect size estimated in a large-scale GWAS and averaged by the *M,* number of SNPs used in the PRS construction.

We followed the same testing paradigm for all PRS models via 10-fold cross validation procedure and a logistic regression framework. The full logistic model includes age, sex, recruitment site, the first 10 PCs, and the PRS; the base model includes all terms except the PRS. We calculated Nagelkerke’s pseudo R^2^ using the DescTools [15] R package and estimated the variance explained on the liability scale using the method described by Lee et al. [16]. We obtained the observed R^2^ by subtracting the base model R^2^ from the full model R^2^; we then converted this to the liability scale using a prevalence of 0.5% and the proportion of cases in LARGE-PD [16,17]. We determined the area under the receiver operator-curve (AUC) using the pROC package [18] in R and predictions generated from the 10 folds using both the full model and the PRS alone. Statistical significance between the AUCs obtained in different models was determined using Delong’s test via the pROC package. We also obtained p-values and the Pseudo R^2^ via the mean of the 10 folds.

To protect against confounding, we repeated the overall analysis by resolving relative pairs via removing one sample as identified using the KING-robust software [19] (testing 2^nd^ and 3^rd^ degree thresholds), by down-sampling the number of Peruvian PD cases, and by including additional external Peruvian controls in our dataset to ensure our results are not being driven by case-control imbalances. We also excluded subjects who were outliers by ancestry. For a previous study[3], the ancestry proportions of LARGE-PD subjects were inferred by merging with 1000 Genomes Project (1KGP) [14] subjects and using the software ADMIXTURE [20] with a K of 5. Potential outliers were identified by selecting the subjects with three times the standard deviation greater or less than the mean of each of the 5 inferred clusters. Principal components were then re-computed with these subjects excluded.

*PRS validation:*

To validate the PD PRS performance, we repeated the GWAS-significant PRS analysis after incorporating external Peruvian controls from the Luo_TB cohort [9]. After imputation, we calculated a PD PRS in all subjects from the TB cohort over the age of 50 using the same set of variants as before and combined this data with LARGE-PD. To calculate principal components, we merged imputed LARGE-PD and Luo_TB data, keeping the intersection of 7,122,988 variants with an imputation R^2^ of 0.9 and a MAF of 0.01 in both datasets. We then performed two rounds of pruning using PLINK’s indep-pairwise algorithm with parameters of 50 SNPs per window, a step of 5 SNPs, and an R^2^ of 0.2, leaving us with ­­­ 341,969 variants for the estimation of PCs and a kinship matrix using PC-AiR [21] and PC-Relate [22]. To evaluate the quality of the merger, we calculated GC Lambda from a GWAS on 766,828 SNPs obtained from the first LD pruning step. We performed the GWAS using a logistic mixed model implemented by the GENESIS R package [23], adjusting for age, sex, the first 10 PCs, and the genetic relationship matrix obtained from PC-Relate. We evaluated the PRS in the same manner as previously described, though in this case the full model included age, sex, the first 10 PCs, recruitment site, and study.

We also tested both the GWAS-significant and the full summary statistics PRS (PRS-full) in an independent cohort of 448 samples (NEUROX_C). Out of the 1040 variants used in the PRS constructed with the full PD summary statistics, 950 were imputed with a minimum imputation R^2^ of 0.8 in NeuroC samples, but only 651 were imputed with a minimum imputation R^2^ of 0.8 in the intersection of the NeuroC and NeuroX samples.

*PD PRS distribution in LARGE-PD*

We visualized the PRS distribution in LARGE-PD and the external Peruvian controls using R. For clustering by PC, we constructed a distance matrix by taking the Euclidean distance of the first two PCs after scaling. Then, we performed k-means clustering via the kmeans function in R, using the Hartigan-Wong algorithm, 5 centers, a maximum of 100 iterations, and 10 random sets. The ancestry of each cluster was then inferred via the mean ancestry proportions of subjects within the cluster obtained using the ADMIXTURE [20] software as described above.

*Characterization of GWAS-significant loci across diverse populations*

We characterized the distribution of the PD PRS across global populations using the high coverage data from the 1000 Genomes Project (1KGP) [14] data. We explored the relationship of admixture with the PD PRS in 1KGP Latinos by utilizing the ancestry proportions estimated with ADMIXTURE as described above and obtaining correlations between each ancestry proportions and the PD PRS using Pearson’s method. Differences in PD PRS distribution across 1KGP super-populations (AFR, AMR, EAS, EUR, SAS) were assessed using the Wilcoxon rank-sum test by using the EUR populations as a reference. To assess differences in risk allele frequencies across super-populations, we created a contingency table for each non-European super-population based on direction of effect and allele frequency. For direction of effect, we counted variants with a positive beta coefficient; for allele frequency, we counted variants with a higher frequency in the given super-population compared to EUR populations. We then tested each contingency table using the Chi-Square test with a single degree of freedom.

*SNCA haplotype analysis: regression model description*

*SNCA* haplotypes containing the variant rs356182 were evaluated in LARGE-PD, a Latino cohort, and IPDGC-EUR, a European-ancestry cohort. In the IPDGC-EUR cohort, the regression model included age, sex, cohort, and the first 5 PCs. In LARGE-PD, the regression model included age, sex, recruitment site, and the first 10 PCs. PCs 1-10 were included in the LARGE-PD models to account for the additional population structure present in an admixed cohort. IPDGC-EUR regression models only included PCs 1-5 since this cohort is comprised of a single continental ancestry.

**Supplemental References**

[1] S.A. Bien, G.L. Wojcik, N. Zubair, C.R. Gignoux, A.R. Martin, J.M. Kocarnik, L.W. Martin, S. Buyske, J. Haessler, R.W. Walker, I. Cheng, M. Graff, L. Xia, N. Franceschini, T. Matise, R. James, L. Hindorff, L. Le Marchand, K.E. North, C.A. Haiman, U. Peters, R.J.F. Loos, C.L. Kooperberg, C.D. Bustamante, E.E. Kenny, C.S. Carlson, Strategies for Enriching Variant Coverage in Candidate Disease Loci on a Multiethnic Genotyping Array, PLoS ONE. 11 (2016). https://doi.org/10.1371/journal.pone.0167758.

[2] W.R. Gibb, A.J. Lees, The relevance of the Lewy body to the pathogenesis of idiopathic Parkinson’s disease, J. Neurol. Neurosurg. Psychiatry. 51 (1988) 745–752. https://doi.org/10.1136/jnnp.51.6.745.

[3] D.P. Loesch, A.R.V.R. Horimoto, K. Heilbron, E.I. Sarihan, M. Inca-Martinez, E. Mason, M. Cornejo-Olivas, L. Torres, P. Mazzetti, C. Cosentino, E. Sarapura-Castro, A. Rivera-Valdivia, A.C. Medina, E. Dieguez, V. Raggio, A. Lescano, V. Tumas, V. Borges, H.B. Ferraz, C.R. Rieder, A. Schumacher-Schuh, B.L. Santos-Lobato, C. Velez-Pardo, M. Jimenez-Del-Rio, F. Lopera, S. Moreno, P. Chana-Cuevas, W. Fernandez, G. Arboleda, H. Arboleda, C.E. Arboleda-Bustos, D. Yearout, C.P. Zabetian, 23andMe Research Team, P. Cannon, T.A. Thornton, T.D. O’Connor, I.F. Mata, Latin American Research Consortium on the Genetics of Parkinson’s Disease (LARGE-PD), Characterizing the Genetic Architecture of Parkinson’s Disease in Latinos, Ann. Neurol. 90 (2021) 353–365. https://doi.org/10.1002/ana.26153.

[4] Ten Years of the International Parkinson Disease Genomics Consortium: Progress and Next Steps, J. Park. Dis. 10 (n.d.) 19–30. https://doi.org/10.3233/JPD-191854.

[5] M.A. Nalls, J. Bras, D.G. Hernandez, M.F. Keller, E. Majounie, A.E. Renton, M. Saad, I. Jansen, R. Guerreiro, S. Lubbe, V. Plagnol, J.R. Gibbs, C. Schulte, N. Pankratz, M. Sutherland, L. Bertram, C.M. Lill, A.L. DeStefano, T. Faroud, N. Eriksson, J.Y. Tung, C. Edsall, N. Nichols, J. Brooks, S. Arepalli, H. Pliner, C. Letson, P. Heutink, M. Martinez, T. Gasser, B.J. Traynor, N. Wood, J. Hardy, A.B. Singleton, International Parkinson’s Disease Genomics Consortium (IPDGC), Parkinson’s Disease meta-analysis consortium, NeuroX, a fast and efficient genotyping platform for investigation of neurodegenerative diseases, Neurobiol. Aging. 36 (2015) 1605.e7–12. https://doi.org/10.1016/j.neurobiolaging.2014.07.028.

[6] C. Blauwendraat, F. Faghri, L. Pihlstrom, J.T. Geiger, A. Elbaz, S. Lesage, J.-C. Corvol, P. May, A. Nicolas, Y. Abramzon, N.A. Murphy, J.R. Gibbs, M. Ryten, R. Ferrari, J. Bras, R. Guerreiro, J. Williams, R. Sims, S. Lubbe, D.G. Hernandez, K.Y. Mok, L. Robak, R.H. Campbell, E. Rogaeva, B.J. Traynor, R. Chia, S.J. Chung, J.A. Hardy, A. Brice, N.W. Wood, H. Houlden, J.M. Shulman, H.R. Morris, T. Gasser, R. Krüger, P. Heutink, M. Sharma, J. Simón-Sánchez, M.A. Nalls, A.B. Singleton, S.W. Scholz, NeuroChip, an updated version of the NeuroX genotyping platform to rapidly screen for variants associated with neurological diseases, Neurobiol. Aging. 57 (2017) 247.e9-247.e13. https://doi.org/10.1016/j.neurobiolaging.2017.05.009.

[7] M. Byrska-Bishop, U.S. Evani, X. Zhao, A.O. Basile, H.J. Abel, A.A. Regier, A. Corvelo, W.E. Clarke, R. Musunuri, K. Nagulapalli, S. Fairley, A. Runnels, L. Winterkorn, E. Lowy-Gallego, T.H.G.S.V. Consortium, P. Flicek, S. Germer, H. Brand, I.M. Hall, M.E. Talkowski, G. Narzisi, M.C. Zody, High coverage whole genome sequencing of the expanded 1000 Genomes Project cohort including 602 trios, BioRxiv. (2021) 2021.02.06.430068. https://doi.org/10.1101/2021.02.06.430068.

[8] D.N. Harris, W. Song, A.C. Shetty, K.S. Levano, O. Cáceres, C. Padilla, V. Borda, D. Tarazona, O. Trujillo, C. Sanchez, M.D. Kessler, M. Galarza, S. Capristano, H. Montejo, P.O. Flores-Villanueva, E. Tarazona-Santos, T.D. O’Connor, H. Guio, Evolutionary genomic dynamics of Peruvians before, during, and after the Inca Empire, Proc. Natl. Acad. Sci. 115 (2018) E6526–E6535. https://doi.org/10.1073/pnas.1720798115.

[9] Y. Luo, S. Suliman, S. Asgari, T. Amariuta, Y. Baglaenko, M. Martínez-Bonet, K. Ishigaki, M. Gutierrez-Arcelus, R. Calderon, L. Lecca, S.R. León, J. Jimenez, R. Yataco, C. Contreras, J.T. Galea, M. Becerra, S. Nejentsev, P.A. Nigrovic, D.B. Moody, M.B. Murray, S. Raychaudhuri, Early progression to active tuberculosis is a highly heritable trait driven by 3q23 in Peruvians, Nat. Commun. 10 (2019) 3765. https://doi.org/10.1038/s41467-019-11664-1.

[10] D. Taliun, D.N. Harris, M.D. Kessler, J. Carlson, Z.A. Szpiech, R. Torres, S.A.G. Taliun, A. Corvelo, S.M. Gogarten, H.M. Kang, A.N. Pitsillides, J. LeFaive, S.-B. Lee, X. Tian, B.L. Browning, S. Das, A.-K. Emde, W.E. Clarke, D.P. Loesch, A.C. Shetty, T.W. Blackwell, A.V. Smith, Q. Wong, X. Liu, M.P. Conomos, D.M. Bobo, F. Aguet, C. Albert, A. Alonso, K.G. Ardlie, D.E. Arking, S. Aslibekyan, P.L. Auer, J. Barnard, R.G. Barr, L. Barwick, L.C. Becker, R.L. Beer, E.J. Benjamin, L.F. Bielak, J. Blangero, M. Boehnke, D.W. Bowden, J.A. Brody, E.G. Burchard, B.E. Cade, J.F. Casella, B. Chalazan, D.I. Chasman, Y.-D.I. Chen, M.H. Cho, S.H. Choi, M.K. Chung, C.B. Clish, A. Correa, J.E. Curran, B. Custer, D. Darbar, M. Daya, M. de Andrade, D.L. DeMeo, S.K. Dutcher, P.T. Ellinor, L.S. Emery, C. Eng, D. Fatkin, T. Fingerlin, L. Forer, M. Fornage, N. Franceschini, C. Fuchsberger, S.M. Fullerton, S. Germer, M.T. Gladwin, D.J. Gottlieb, X. Guo, M.E. Hall, J. He, N.L. Heard-Costa, S.R. Heckbert, M.R. Irvin, J.M. Johnsen, A.D. Johnson, R. Kaplan, S.L.R. Kardia, T. Kelly, S. Kelly, E.E. Kenny, D.P. Kiel, R. Klemmer, B.A. Konkle, C. Kooperberg, A. Köttgen, L.A. Lange, J. Lasky-Su, D. Levy, X. Lin, K.-H. Lin, C. Liu, R.J.F. Loos, L. Garman, R. Gerszten, S.A. Lubitz, K.L. Lunetta, A.C.Y. Mak, A. Manichaikul, A.K. Manning, R.A. Mathias, D.D. McManus, S.T. McGarvey, J.B. Meigs, D.A. Meyers, J.L. Mikulla, M.A. Minear, B.D. Mitchell, S. Mohanty, M.E. Montasser, C. Montgomery, A.C. Morrison, J.M. Murabito, A. Natale, P. Natarajan, S.C. Nelson, K.E. North, J.R. O’Connell, N.D. Palmer, N. Pankratz, G.M. Peloso, P.A. Peyser, J. Pleiness, W.S. Post, B.M. Psaty, D.C. Rao, S. Redline, A.P. Reiner, D. Roden, J.I. Rotter, I. Ruczinski, C. Sarnowski, S. Schoenherr, D.A. Schwartz, J.-S. Seo, S. Seshadri, V.A. Sheehan, W.H. Sheu, M.B. Shoemaker, N.L. Smith, J.A. Smith, N. Sotoodehnia, A.M. Stilp, W. Tang, K.D. Taylor, M. Telen, T.A. Thornton, R.P. Tracy, D.J. Van Den Berg, R.S. Vasan, K.A. Viaud-Martinez, S. Vrieze, D.E. Weeks, B.S. Weir, S.T. Weiss, L.-C. Weng, C.J. Willer, Y. Zhang, X. Zhao, D.K. Arnett, A.E. Ashley-Koch, K.C. Barnes, E. Boerwinkle, S. Gabriel, R. Gibbs, K.M. Rice, S.S. Rich, E.K. Silverman, P. Qasba, W. Gan, NHLBI Trans-Omics for Precision Medicine (TOPMed) Consortium, G.J. Papanicolaou, D.A. Nickerson, S.R. Browning, M.C. Zody, S. Zöllner, J.G. Wilson, L.A. Cupples, C.C. Laurie, C.E. Jaquish, R.D. Hernandez, T.D. O’Connor, G.R. Abecasis, Sequencing of 53,831 diverse genomes from the NHLBI TOPMed Program, Nature. 590 (2021) 290–299. https://doi.org/10.1038/s41586-021-03205-y.

[11] M.H. Kowalski, H. Qian, Z. Hou, J.D. Rosen, A.L. Tapia, Y. Shan, D. Jain, M. Argos, D.K. Arnett, C. Avery, K.C. Barnes, L.C. Becker, S.A. Bien, J.C. Bis, J. Blangero, E. Boerwinkle, D.W. Bowden, S. Buyske, J. Cai, M.H. Cho, S.H. Choi, H. Choquet, L.A. Cupples, M. Cushman, M. Daya, P.S. de Vries, P.T. Ellinor, N. Faraday, M. Fornage, S. Gabriel, S.K. Ganesh, M. Graff, N. Gupta, J. He, S.R. Heckbert, B. Hidalgo, C.J. Hodonsky, M.R. Irvin, A.D. Johnson, E. Jorgenson, R. Kaplan, S.L.R. Kardia, T.N. Kelly, C. Kooperberg, J.A. Lasky-Su, R.J.F. Loos, S.A. Lubitz, R.A. Mathias, C.P. McHugh, C. Montgomery, J.-Y. Moon, A.C. Morrison, N.D. Palmer, N. Pankratz, G.J. Papanicolaou, J.M. Peralta, P.A. Peyser, S.S. Rich, J.I. Rotter, E.K. Silverman, J.A. Smith, N.L. Smith, K.D. Taylor, T.A. Thornton, H.K. Tiwari, R.P. Tracy, T. Wang, S.T. Weiss, L.-C. Weng, K.L. Wiggins, J.G. Wilson, L.R. Yanek, S. Zöllner, K.E. North, P.L. Auer, N.T.-O. for P.M. (TOPMed) Consortium, Topm.H.& H.W. Group, L.M. Raffield, A.P. Reiner, Y. Li, Use of >100,000 NHLBI Trans-Omics for Precision Medicine (TOPMed) Consortium whole genome sequences improves imputation quality and detection of rare variant associations in admixed African and Hispanic/Latino populations, PLOS Genet. 15 (2019) e1008500. https://doi.org/10.1371/journal.pgen.1008500.

[12] M.A. Nalls, C. Blauwendraat, C.L. Vallerga, K. Heilbron, S. Bandres-Ciga, D. Chang, M. Tan, D.A. Kia, A.J. Noyce, A. Xue, J. Bras, E. Young, R. von Coelln, J. Simón-Sánchez, C. Schulte, M. Sharma, L. Krohn, L. Pihlstrøm, A. Siitonen, H. Iwaki, H. Leonard, F. Faghri, J.R. Gibbs, D.G. Hernandez, S.W. Scholz, J.A. Botia, M. Martinez, J.-C. Corvol, S. Lesage, J. Jankovic, L.M. Shulman, M. Sutherland, P. Tienari, K. Majamaa, M. Toft, O.A. Andreassen, T. Bangale, A. Brice, J. Yang, Z. Gan-Or, T. Gasser, P. Heutink, J.M. Shulman, N.W. Wood, D.A. Hinds, J.A. Hardy, H.R. Morris, J. Gratten, P.M. Visscher, R.R. Graham, A.B. Singleton, A.D. Adarmes-Gómez, M. Aguilar, A. Aitkulova, V. Akhmetzhanov, R.N. Alcalay, I. Alvarez, V. Alvarez, S. Bandres-Ciga, F.J. Barrero, J.A. Bergareche Yarza, I. Bernal-Bernal, K. Billingsley, C. Blauwendraat, M. Blazquez, M. Bonilla-Toribio, J.A. Botía, M.T. Boungiorno, J. Bras, A. Brice, K. Brockmann, V. Bubb, D. Buiza-Rueda, A. Cámara, F. Carrillo, M. Carrión-Claro, D. Cerdan, V. Chelban, J. Clarimón, C. Clarke, Y. Compta, M.R. Cookson, J.-C. Corvol, D.W. Craig, F. Danjou, M. Diez-Fairen, O. Dols-Icardo, J. Duarte, R. Duran, F. Escamilla-Sevilla, V. Escott-Price, M. Ezquerra, F. Faghri, C. Feliz, M. Fernández, R. Fernández-Santiago, S. Finkbeiner, T. Foltynie, Z. Gan-Or, C. Garcia, P. García-Ruiz, T. Gasser, J.R. Gibbs, M.J. Gomez Heredia, P. Gómez-Garre, M.M. González, I. Gonzalez-Aramburu, S. Guelfi, R. Guerreiro, J. Hardy, S. Hassin-Baer, D.G. Hernandez, P. Heutink, J. Hoenicka, P. Holmans, H. Houlden, J. Infante, H. Iwaki, S. Jesús, A. Jimenez-Escrig, G. Kaishybayeva, R. Kaiyrzhanov, A. Karimova, D.A. Kia, K.J. Kinghorn, S. Koks, L. Krohn, J. Kulisevsky, M.A. Labrador-Espinosa, H.L. Leonard, S. Lesage, P. Lewis, J.L. Lopez-Sendon, R. Lovering, S. Lubbe, C. Lungu, D. Macias, K. Majamaa, C. Manzoni, J. Marín, J. Marinus, M.J. Marti, M. Martinez, I. Martínez Torres, J.C. Martínez-Castrillo, M. Mata, N.E. Mencacci, C. Méndez-del-Barrio, B. Middlehurst, A. Mínguez, P. Mir, K.Y. Mok, H.R. Morris, E. Muñoz, M.A. Nalls, D. Narendra, A.J. Noyce, O.O. Ojo, N.U. Okubadejo, A.G. Pagola, P. Pastor, F. Perez Errazquin, T. Periñán-Tocino, L. Pihlstrom, H. Plun-Favreau, J. Quinn, L. R’Bibo, X. Reed, E.M. Rezola, M. Rizig, P. Rizzu, L. Robak, A.S. Rodriguez, G.A. Rouleau, J. Ruiz-Martínez, C. Ruz, M. Ryten, D. Sadykova, S.W. Scholz, S. Schreglmann, C. Schulte, M. Sharma, C. Shashkin, J.M. Shulman, M. Sierra, A. Siitonen, J. Simón-Sánchez, A.B. Singleton, E. Suarez-Sanmartin, P. Taba, C. Tabernero, M.X. Tan, J.P. Tartari, C. Tejera-Parrado, M. Toft, E. Tolosa, D. Trabzuni, F. Valldeoriola, J.J. van Hilten, K. Van Keuren-Jensen, L. Vargas-González, L. Vela, F. Vives, N. Williams, N.W. Wood, N. Zharkinbekova, Z. Zharmukhanov, E. Zholdybayeva, A. Zimprich, P. Ylikotila, L.M. Shulman, R. von Coelln, S. Reich, J. Savitt, M. Agee, B. Alipanahi, A. Auton, R.K. Bell, K. Bryc, S.L. Elson, P. Fontanillas, N.A. Furlotte, K.E. Huber, B. Hicks, E.M. Jewett, Y. Jiang, A. Kleinman, K.-H. Lin, N.K. Litterman, J.C. McCreight, M.H. McIntyre, K.F. McManus, J.L. Mountain, E.S. Noblin, C.A.M. Northover, S.J. Pitts, G.D. Poznik, J.F. Sathirapongsasuti, J.F. Shelton, S. Shringarpure, C. Tian, J. Tung, V. Vacic, X. Wang, C.H. Wilson, T. Anderson, S. Bentley, J. Dalrymple-Alford, J. Fowdar, J. Gratten, G. Halliday, A.K. Henders, I. Hickie, I. Kassam, M. Kennedy, J. Kwok, S. Lewis, G. Mellick, G. Montgomery, J. Pearson, T. Pitcher, J. Sidorenko, P.A. Silburn, C.L. Vallerga, P.M. Visscher, L. Wallace, N.R. Wray, A. Xue, J. Yang, F. Zhang, Identification of novel risk loci, causal insights, and heritable risk for Parkinson’s disease: a meta-analysis of genome-wide association studies, Lancet Neurol. 18 (2019) 1091–1102. https://doi.org/10.1016/S1474-4422(19)30320-5.

[13] S.W. Choi, P.F. O’Reilly, PRSice-2: Polygenic Risk Score software for biobank-scale data, GigaScience. 8 (2019). https://doi.org/10.1093/gigascience/giz082.

[14] T. 1000 G.P. Consortium, A global reference for human genetic variation, Nature. 526 (2015) 68. https://doi.org/10.1038/nature15393.

[15] A. Signorell, K. Aho, A. Alfons, N. Anderegg, T. Aragon, A. Arppe, A. Baddeley, K. Barton, B. Bolker, H.W. Borchers, F. Caeiro, S. Champely, D. Chessel, L. Chhay, C. Cummins, M. Dewey, H.C. Doran, S. Dray, C. Dupont, D. Eddelbuettel, J. Enos, C. Ekstrom, M. Elff, K. Erguler, R.W. Farebrother, J. Fox, R. Francois, M. Friendly, T. Galili, M. Gamer, J.L. Gastwirth, Y.R. Gel, V. Gegzna, J. Gross, G. Grothendieck, F.E.H. Jr, R. Heiberger, M. Hoehle, C.W. Hoffmann, S. Hojsgaard, T. Hothorn, M. Huerzeler, W.W. Hui, P. Hurd, R.J. Hyndman, P.J.V. Iglesias, C. Jackson, M. Kohl, M. Korpela, M. Kuhn, D. Labes, D.T. Lang, F. Leisch, J. Lemon, D. Li, M. Maechler, A. Magnusson, D. Malter, G. Marsaglia, J. Marsaglia, A. Matei, D. Meyer, W. Miao, G. Millo, Y. Min, D. Mitchell, F. Mueller, M. Naepflin, D. Navarro, H. Nilsson, K. Nordhausen, D. Ogle, H. Ooi, N. Parsons, S. Pavoine, T. Plate, R. Rapold, W. Revelle, T. Rinker, B.D. Ripley, C. Rodriguez, N. Russell, N. Sabbe, V.E. Seshan, G. Snow, M. Smithson, K. Soetaert, W.A. Stahel, A. Stephenson, M. Stevenson, R. Stubner, M. Templ, T. Therneau, Y. Tille, A. Trapletti, J. Ulrich, K. Ushey, J. VanDerWal, B. Venables, J. Verzani, G.R. Warnes, S. Wellek, H. Wickham, R.R. Wilcox, P. Wolf, D. Wollschlaeger, J. Wood, Y. Wu, T. Yee, A. Zeileis, DescTools: Tools for Descriptive Statistics, 2019. https://CRAN.R-project.org/package=DescTools (accessed December 9, 2019).

[16] S.H. Lee, M.E. Goddard, N.R. Wray, P.M. Visscher, A better coefficient of determination for genetic profile analysis, Genet. Epidemiol. 36 (2012) 214–224. https://doi.org/10.1002/gepi.21614.

[17] N.R. Wray, J. Yang, M.E. Goddard, P.M. Visscher, The Genetic Interpretation of Area under the ROC Curve in Genomic Profiling, PLoS Genet. 6 (2010). https://doi.org/10.1371/journal.pgen.1000864.

[18] X. Robin, N. Turck, A. Hainard, N. Tiberti, F. Lisacek, J.-C. Sanchez, M. Müller, pROC: an open-source package for R and S+ to analyze and compare ROC curves, BMC Bioinformatics. 12 (2011) 77. https://doi.org/10.1186/1471-2105-12-77.

[19] A. Manichaikul, J.C. Mychaleckyj, S.S. Rich, K. Daly, M. Sale, W.-M. Chen, Robust relationship inference in genome-wide association studies, Bioinformatics. 26 (2010) 2867–2873. https://doi.org/10.1093/bioinformatics/btq559.

[20] D.H. Alexander, J. Novembre, K. Lange, Fast model-based estimation of ancestry in unrelated individuals, Genome Res. 19 (2009) 1655–1664. https://doi.org/10.1101/gr.094052.109.

[21] M.P. Conomos, M. Miller, T. Thornton, Robust Inference of Population Structure for Ancestry Prediction and Correction of Stratification in the Presence of Relatedness, Genet. Epidemiol. 39 (2015) 276–293. https://doi.org/10.1002/gepi.21896.

[22] M.P. Conomos, A.P. Reiner, B.S. Weir, T.A. Thornton, Model-free Estimation of Recent Genetic Relatedness, Am. J. Hum. Genet. 98 (2016) 127–148. https://doi.org/10.1016/j.ajhg.2015.11.022.

[23] S.M. Gogarten, T. Sofer, H. Chen, C. Yu, J.A. Brody, T.A. Thornton, K.M. Rice, M.P. Conomos, Genetic association testing using the GENESIS R/Bioconductor package, Bioinformatics. 35 (2019) 5346–5348. https://doi.org/10.1093/bioinformatics/btz567.

**Supplemental Figures**

**Supplementary Figure 1: Performance of PD PRS in LARGE-PD**

**A. B.**

**A:** Odds ratios of LARGE-PD subjects with a PD PRS in quintiles 2 through 5 compared to quintile 1 (orange) and odds ratios of LARGE-PD subjects plus the addition of external Peruvian controls (blue). **B:** Plots of the receiver operator curve (ROC) when predicting PD status using the PD PRS alone for Peruvian LARGE-PD subjects (silver), all of LARGE-PD (red), non-Peruvian LARGE-PD subjects (light blue), and LARGE-PD plus 440 external Peruvian controls (light blue). ROC curves were generated using the pROC package in R and the PD PRS consists of only independent GWAS-significant variants.

**Supplementary Figure 2: AUC in NEUROX_C cohort of Latino subjects.**

**A. B.**

**A:** Receiver-operator curve (ROC) of the PD PRS constructed using only GWAS-significant variants in the NeuroX_C cohort supplied by the IPDGC. **B:** ROC of the PD PRS constructed using the full PD GWAS summary statistics from Nalls et al. 2019.

**Supplementary Figure 3: PD PRS distribution in controls only**

Principal components (PCs) of PD controls and density plots of the PD PRS distribution. **A.** Plot of PC 1 versus PC2 colored by PC-derived clusters using k-means clustering. **B:** Distribution of the PD PRS colored by PC-derived clusters. We used ancestry proportions estimated by ADMIXURE to characterize clusters.

**Supplementary Figure 4: PD PRS by** **Ancestry in 1KGP Latinos**

**A:** Ancestry proportions of 1KGP Latinos as estimated by ADMIXTURE. **B:** Scatterplot of PD PRS versus European ancestry proportion with the regression line in blue (95% confidence interval in grey). **C:** Scatterplot of PD PRS versus Native American ancestry proportion with the regression line in blue (95% confidence interval in grey). **D:** Scatterplot of PD PRS versus African ancestry proportion with the regression line in blue (95% confidence interval in grey).

**Supplementary Figure 5: PRS distribution by 1000 Genomes Project Super-Population**

Distribution in the 1000 Genomes Project (1KGP) of the scaled PD PRS constructed using GWAS-significant variants in the 1KGP-defined super-populations of AFR (African), AMR (Admixed American i.e., Latinos), SAS (South Asian), EAS (East Asian), and EUR (European).

**Supplementary Figure 6: PD risk allele frequencies in African versus European populations**

Scatter plot of PD risk allele frequencies in African populations (X-axis) versus European populations (y-axis) from the 1000 Genomes Project. Variants with a positive direction of effect were labeled as risk (orange) while variants with a negative direction of effect were labeled protective (blue). Variants above the black line are higher in frequency in European populations, while variants below the black line are more frequent in African populations. Note the preponderance of risk variants with a higher frequency in European populations.

**Supplementary Figure 7: PD PRS and Age at Onset**

**A. All Subjects**

**B. Cases Only**

Kaplan-Meier curves of age at PD onset stratified by GWAS-significant PRS quintile in all subjects (**A**) and in cases only (**B**) generated using the survival package in R. For controls, age at analysis was used. Cases with an age at onset less than 18 years were excluded.

**Supplementary Figure 8: Haplotype Network of rs356182 haplotypes.**

Haplotype network of haplotypes from 1KGP, LARGE-PD, and an IPDGC PD cohort of European descent as constructed using the TCS network algorithm and POPArt. Note the presence of two distinct G-allele haplotype clusters which were separated by A-allele haplotypes.

**Supplementary Figure 9: Heatmap of shared alleles between common rs356182 haplotypes**

Heatmap of the proportion of shared alleles between common rs356182 haplotypes. For example, the haplotypes hap1 and hap9 share 0.14 or 14% of their alleles, including rs356182. Haplotypes were extracted from a 33.6 kb region from the merged 1000 Genomes Project, Peruvian Genome Project, LARGE-PD, and IPDGC data.

**Supplemental Tables**

**Supplementary Table 1: Cohort Description**

| Cohort | N (N Unrel.; N Cases) | Mean (SD) Age | Sex | Data Type | Recruitment Country |
| --- | --- | --- | --- | --- | --- |
| LARGE-PD | 1497 (1447; 807) | 59.3 (13.9) | 44.3% male | Genotyped + imputed | Peru, Brazil, Colombia, Chile, Uruguay |
| External Controls (Luo_TB) | 440 (429; 0) | 62.65 (9.13) | 46.4% male | Genotyped + imputed | Peru |
| IPDGC-Latino (NeuroX_C) | 448 (431; 225) | Not known | 49.55% male | Genotyped + imputed | USA (Latino/Hispanic) |
| IPDGC-European | 2446 (2446; 715) | 73 (18.4) | 54.2% male | Sequenced | USA, UK (European-ancestry) |
| Peruvian Genome Project (PGP) | 150 (150; 0) | NA | NA | Sequenced | Peru |
| 1000 Genomes Project (1KGP) | 2504 (2504; 0) | NA | 49.2% male | Sequenced | Multiple |

N (N Cases): Number of subjects (Number of unrelated subjects; Number of cases). Mean (SD) Age: Mean and SD of age at analysis. Sex: Proportion of the cohort that is male. Data Type: Indicates type of genetic data, i.e., sequenced or genotyped. Recruitment Country: Primary country for cohort recruitment.

**Supplementary Table 2: PRS by 1000 Genomes Super-Population**

| **POP** | **MEAN** | **SD** | **PVAL** |
| --- | --- | --- | --- |
| **EUR** | 0.17 | 0.55 | NA |
| **EAS** | 0.4 | 0.47 | 3.49x10^-12^ |
| **AMR** | 0.08 | 0.57 | 0.02 |
| **SAS** | 0.23 | 0.54 | 0.05 |
| **AFR** | -1.21 | 0.46 | 2.02x10^-169^ |

POP: 1KGP super-population label. MEAN: mean PRS. SD: standard deviation of the PRS. PVAL: p-value of the Wilcoxon test with European subjects as reference.

**Supplementary Table 3: PD PRS in Peruvian Populations**

| **GROUP** | **N** | **MEAN** | **SD** | **P-VALUE** |
| --- | --- | --- | --- | --- |
| **PEL** | 85 | 0.581 | 0.562 | NA |
| **PERUVIAN_CASES** | 437 | 0.865 | 0.508 | 2.03x10^-5^ |
| **PERUVIAN_CONTROLS** | 233 | 0.542 | 0.519 | 0.6058 |
| **PUNO** | 45 | 0.500 | 0.509 | 0.3679 |
| **TB_ALL** | 4009 | 0.641 | 0.497 | 0.2764 |
| **TB_CONTROLS** | 440 | 0.661 | 0.510 | 0.2169 |
| **PGP-ALL** | 150 | 0.679 | 0.462 | 0.1658 |
| **PGP-CHOPCCAS** | 30 | 0.880 | 0.499 | 0.02204 |
| **PGP-CUSCO** | 16 | 0.624 | 0.450 | 0.7273 |
| **PGP-IQUITOS** | 16 | 0.633 | 0.470 | 0.561 |
| **PGP-MATZES** | 12 | 0.802 | 0.385 | 0.1348 |
| **PGP-MOCHES** | 30 | 0.599 | 0.411 | 0.8635 |
| **PGP-TRUJILLO** | 16 | 0.638 | 0.609 | 0.5928 |
| **PGP-UROS** | 30 | 0.585 | 0.378 | 0.9467 |

GROUP: group label. N: number of subjects. MEAN: mean of raw PRS. SD: standard deviation of PRS. P-VALUE: p-value of PRS using the Wilcoxon rank sum test and PEL subjects as a reference.

**Supplementary Table 4: Cox regression results**

| QUINTILE | HR | 95% CI | PVALUE |
| --- | --- | --- | --- |
| CASES ONLY | | | |
| 2 | 1.10 | 0.832- 1.44 | 0.515 |
| 3 | 1.14 | 0.879 - 1.48 | 0.324 |
| 4 | 1.13 | 0.878 - 1.46 | 0.341 |
| 5 | 1.45 | 1.17 - 1.91 | 0.003 |
| CASES + CONTROLS | | | |
| 2 | 1.28 | 0.984 - 1.68 | 0.096 |
| 3 | 1.43 | 1.11 - 1.85 | 0.007 |
| 4 | 1.60 | 1.24 - 2.06 | 2.55x10^-04^ |
| 5 | 2.29 | 1.79 - 2.93 | 3.41x10^-11^ |

QUINTILE: quintile of the PD PRS. HR: hazard ratio. 95% CI: 95% confidence interval of the hazard ratio. PVALUE: p-value of the hazard ratio.

**Supplementary Table 5: Leave-one-out PD PRS**

| SNP | R2 | DIFF |
| --- | --- | --- |
| rs35749011 | 0.0212 | -3.43% |
| rs76763715 | 0.0218 | -0.64% |
| rs11578699 | 0.0220 | 0.12% |
| rs823118 | 0.0223 | 1.34% |
| rs11557080 | 0.0226 | 3.06% |
| rs4653767 | 0.0220 | -0.04% |
| rs10797576 | 0.0217 | -1.21% |
| rs57891859 | 0.0222 | 1.01% |
| rs1474055 | 0.0217 | -1.43% |
| rs73038319 | 0.0217 | -1.24% |
| rs6808178 | 0.0217 | -1.46% |
| rs12497850 | 0.0218 | -0.89% |
| rs55961674 | 0.0214 | -2.48% |
| rs1450522 | 0.0216 | -1.81% |
| rs10513789 | 0.0212 | -3.40% |
| rs873786 | 0.0219 | -0.55% |
| rs34311866 | 0.0219 | -0.25% |
| rs4698412 | 0.0216 | -1.66% |
| rs4101061 | 0.0218 | -0.74% |
| rs6854006 | 0.0225 | 2.29% |
| rs356182 | 0.0177 | -19.60% |
| rs5019538 | 0.0222 | 1.08% |
| rs13117519 | 0.0218 | -0.78% |
| rs62333164 | 0.0220 | -0.07% |
| rs1867598 | 0.0209 | -4.83% |
| rs11950533 | 0.0223 | 1.53% |
| rs4140646 | 0.0217 | -1.17% |
| rs9261484 | 0.0211 | -3.81% |
| rs112485576 | 0.0214 | -2.43% |
| rs12528068 | 0.0222 | 1.23% |
| rs997368 | 0.0220 | 0.17% |
| rs75859381 | 0.0212 | -3.35% |
| rs199351 | 0.0221 | 0.72% |
| rs1293298 | 0.0219 | -0.38% |
| rs620513 | 0.0217 | -1.44% |
| rs2280104 | 0.0213 | -3.22% |
| rs2086641 | 0.0219 | -0.14% |
| rs13294100 | 0.0220 | -0.10% |
| rs10756907 | 0.0217 | -1.05% |
| rs6476434 | 0.0225 | 2.40% |
| rs896435 | 0.0216 | -1.69% |
| rs10748818 | 0.0222 | 1.22% |
| rs72840788 | 0.0220 | 0.19% |
| rs117896735 | 0.0209 | -5.04% |
| rs7938782 | 0.0216 | -1.81% |
| rs12283611 | 0.0218 | -0.96% |
| rs3802920 | 0.0222 | 1.12% |
| rs76904798 | 0.0227 | 3.19% |
| rs34637584 | 0.0218 | -0.97% |
| rs7134559 | 0.0216 | -1.91% |
| rs10847864 | 0.0212 | -3.33% |
| rs11610045 | 0.0219 | -0.40% |
| rs9568188 | 0.0214 | -2.48% |
| rs4771268 | 0.0222 | 1.06% |
| rs12147950 | 0.0218 | -0.89% |
| rs11158026 | 0.0221 | 0.40% |
| rs3742785 | 0.0216 | -1.72% |
| rs979812 | 0.0214 | -2.82% |
| rs2251086 | 0.0213 | -3.24% |
| rs11150601 | 0.0208 | -5.39% |
| rs6500328 | 0.0221 | 0.61% |
| rs3104783 | 0.0220 | 0.14% |
| rs10221156 | 0.0216 | -1.64% |
| rs12600861 | 0.0219 | -0.19% |
| rs12951632 | 0.0215 | -2.29% |
| rs2269906 | 0.0219 | -0.49% |
| rs850738 | 0.0220 | 0.02% |
| rs62053943 | 0.0216 | -1.52% |
| rs117615688 | 0.0202 | -8.05% |
| rs11658976 | 0.0218 | -0.61% |
| rs61169879 | 0.0218 | -0.58% |
| rs1941685 | 0.0219 | -0.27% |
| rs12456492 | 0.0225 | 2.42% |
| rs8087969 | 0.0222 | 1.15% |
| rs55818311 | 0.0222 | 1.23% |
| rs77351827 | 0.0219 | -0.32% |
| rs2248244 | 0.0211 | -4.20% |

SNP: variant removed from PRS. R2: R^2^ on the liability scale of PRS after removal of SNP. DIFF: Percent difference in R^2^ on the liability scale of full 77 variant PD PRS

**Supplementary table 6: SNCA haplotype blocks in select populations**

| **POPULATION** | **COHORT** | **SIZE (KB)** | **NSNP** |
| --- | --- | --- | --- |
| **GIH** | 1KGP-SAS | 0.029 | 2 |
| **STU** | 1KGP-SAS | 0.029 | 2 |
| **FIN** | 1KGP-EUR | 0.029 | 2 |
| **CEU** | 1KGP-EUR | 0 | 0 |
| **CLM** | 1KGP-AMR | 0.029 | 2 |
| **PEL** | 1KGP-AMR | 0.303 | 3 |
| **ASW** | 1KGP-AFR | 0 | 0 |
| **YRI** | 1KGP-AFR | 0 | 0 |
| **CHB** | 1KGP-EAS | 68.573 | 87 |
| **JPT** | 1KGP-EAS | 12.424 | 18 |
| **CHOPCCAS** | PGP | 0.029 | 2 |
| **CUSCO** | PGP | 0 | 0 |
| **IQUITOS** | PGP | 96.637 | 131 |
| **MATZES** | PGP | 108.851 | 187 |
| **MOCHES** | PGP | 0 | 0 |
| **TRUJILLO** | PGP | 0 | 0 |
| **UROS** | PGP | 2.155 | 4 |
| **IPDGC_CONTROL** | IPDGC | 19.504 | 16 |
| **IPDGC_CASE** | IPDGC | 0.029 | 2 |
| **LARGEPD_CONTROL** | LARGEPD | 0.303 | 3 |
| **LARGEPD_CASE** | LARGEPD | 42.802 | 40 |
| **Chile** | LARGEPD | 0 | 0 |
| **Brazil_PortoAlegre** | LARGEPD | 0 | 0 |
| **Brazil_SaoPaolo** | LARGEPD | 0 | 0 |
| **Brazil_RibeiraoPreto** | LARGEPD | 0.029 | 2 |
| **Colombia_Bogota** | LARGEPD | 0 | 0 |
| **Colombia_Medellin** | LARGEPD | 0.327 | 3 |
| **Peru (Lima)** | LARGEPD | 33.628 | 57 |
| **Peru_Puno** | LARGEPD | 108.622 | 131 |
| **Uruguay** | LARGEPD | 0.029 | 2 |

POPULATION: population label. COHORT: cohort label. SIZE: haplotype size in kilobases. NSNP: number of SNPs in the haplotype.

**Supplementary table 7: IPDGC rs356182 haplotype analysis**

| **hapID** | **ALLELE** | **FREQ** | **FREQ**  **CASES** | **FREQ**  **CONT** | **BETA**  **(SE)** | **PVAL**  **(ADJ)** | **P_LRT** | **CONC** |
| --- | --- | --- | --- | --- | --- | --- | --- | --- |
| **hap1** | G | 0.28 | 0.3 | 0.26 | 0.36  (0.1) | 0.01  (0.089) | 0.29 | TRUE |
| **hap10** | A | 0.01 | 0.01 | 0.01 | 1.56  (1.1) | 0.14  (1) | . | FALSE |
| **hap2** | A | 0.48 | 0.44 | 0.5 | -0.5  (0.1) | 1.75x10^-4^  (0.001) | 0.15 | TRUE |
| **hap3** | A | 0.05 | 0.05 | 0.05 | -0.1  (0.3) | 0.71  (1) | . | TRUE |
| **hap4** | A | 0.04 | 0.02 | 0.04 | -0.6  (0.4) | 0.13  (0.91) | . | TRUE |
| **hap6** | G | 0.04 | 0.06 | 0.04 | 0.42  (0.3) | 0.2  (1) | . | TRUE |
| **hap9** | G | 0.03 | 0.03 | 0.02 | 0.68  (0.5) | 0.15  (1) | . | TRUE |

hapID: haplotype ID. ALLELE: A or G allele for rs356182. G is the risk allele. FREQ: frequency of the haplotype. FREQ CASES: frequency of the haplotype in cases. FREQ CONT: frequency of the haplotype in controls. BETA (SE): effect size estimated in logistic regression model and standard error of the beta. PVAL (ADJ): p-value of the beta and adjusted p-value after correcting for number of haplotypes tested. P_LRT: p-value from likelihood ratio test evaluating whether inclusion of haplotype information improves models with rs356182 genotype status. CONC: Concordance of the direction of effect with rs356182 allele status.

**Supplementary table 8: LARGE-PD rs356182 haplotype analysis**

| **hapID** | **ALLELE** | **FREQ** | **FREQ**  **CASES** | **FREQ**  **CONT** | **BETA**  **(SE)** | **PVAL**  **(ADJ)** | **P_LRT (ADJ)** | **CONC** |
| --- | --- | --- | --- | --- | --- | --- | --- | --- |
| **hap1** | G | 0.16 | 0.16 | 0.16 | 0.12  (0.12) | 0.287  (1) | . | TRUE |
| **hap11** | A | 0.11 | 0.09 | 0.14 | -0.81  (0.14) | 6.04x10^-9^  (4.83x10^-8^) | 2.65x10^-5^ (7.96x10^-5^) | TRUE |
| **hap2** | A | 0.33 | 0.3 | 0.36 | -0.14  (0.09) | 0.118  (0.944) | . | TRUE |
| **hap22** | A | 0.01 | 0.01 | 0.01 | 0.32  (0.4) | 0.418  (1) | . | FALSE |
| **hap3** | A | 0.03 | 0.03 | 0.03 | 0.42  (0.26) | 0.112  (0.898) | . | TRUE |
| **hap4** | A | 0.02 | 0.01 | 0.02 | -0.58  (0.36) | 0.106  (0.852) | . | TRUE |
| **hap6** | G | 0.01 | 0.02 | 0.01 | 1.18  (0.43) | 0.006  (0.049) | 0.039 (0.117) | TRUE |
| **hap9** | G | 0.24 | 0.3 | 0.17 | 0.5  (0.11) | 4.47x10^-6^  (3.59x10^-5^) | 0.076 (0.228) | TRUE |

hapID: haplotype ID. ALLELE: A or G allele for rs356182. G is the risk allele. FREQ: frequency of the haplotype. FREQ CASES: frequency of the haplotype in cases. FREQ CONT: frequency of the haplotype in controls. BETA (SE): effect size estimated in logistic regression model and standard error of the beta. PVAL (ADJ): p-value of the beta and adjusted p-value after correcting for number of haplotypes tested. P_LRT (ADJ): p-value from likelihood ratio test evaluating whether inclusion of haplotype information improves models with rs356182 genotype status and p-value adjusted for multiple testing. CONC: Concordance of the direction of effect with rs356182 allele status.
